# Four-dimensional ultrasound guidance during epidural anaesthesia

**DOI:** 10.1007/s40477-014-0150-1

**Published:** 2014-12-04

**Authors:** Alexey G. Voloshin

**Affiliations:** Pain Management Centre, Medical Center “Petrovskie vorota”, 1-st Kolobovsky Lane, 4, 127051 Moscow, Russian Federation

**Keywords:** Anaesthetics, Epidural, Ultrasonography, Interventional, 3-Dimensional, Ultrasound

## Abstract

**Background:**

Four-dimensional (4D) ultrasound scanning (3D real-time mode) can improve the orientation of the anatomy of the area of interest and navigation by controlling the needle position. The objectives of this study were to identify the optimal technique for navigation and to assess clinically the efficacy of 4D ultrasound navigation for epidural anaesthesia at lower thoracic and lumbar levels.

**Design:**

Single-centre case series study was performed.

**Methods:**

Sixteen patients were included. First, conventional 2D scanning was performed, followed by 4D reconstruction, and the basic tissues with high acoustic impedance (bone structures) and available acoustic windows were determined. Movement of the needle was controlled on the sagittal plane in 2D mode and at the same time in 4D mode (3D real-time mode). To improve the visibility of the needle, the 3D reconstruction was rotated during manipulation.

**Results:**

The 4D scanning mode provided 100 % visibility of compact bone tissues and 93 % visibility of the posterior complex. Needle visualisation strongly depended on the rotation of the reconstructed image with the sensor remaining motionless. The needle was redirected in one patient (7 %) because it was in contact with the vertebral lamina. Dilation of the epidural space during saline injection was observed in five patients (36 %). A change in the puncture level was not required any patients; no complications associated with epidural puncture were observed.

**Conclusions:**

Ultrasound navigation in 4D could improve epidural anaesthesia due to the enhanced spatial orientation of the operator. The technique of “position contrast” should be used for reliable needle visualisation.

**Electronic supplementary material:**

The online version of this article (doi:10.1007/s40477-014-0150-1) contains supplementary material, which is available to authorized users.

## Sommario

**Scopo:**

L’ecografia in quattro dimensioni (4D) (modalità 3D in tempo reale) può migliorare l’orientamento anatomico nella zona d’interesse e la navigazione, controllando la posizione dell’ago. Gli obiettivi di questo studio sono stati di identificare la tecnica ottimale per la navigazione e valutare l’efficacia clinica della navigazione ecografica 4D per l’anestesia epidurale a livello toracico e lombare inferiore. Sono stati studiati una serie di casi di un singolo centro.

**Materiali e Metodi:**

Sedici pazienti sono stati inclusi nello studio. Inizialmente, è stato eseguito un esame convenzionale 2D, seguito da ricostruzione 4D e sono stati individuati tessuti con alta impedenza acustica di base (strutture ossee) e finestre acustiche disponibili. Il movimento dell’ago era controllato sul piano sagittale in modalità 2D e allo stesso tempo in modalità 4D (modalità 3D in tempo reale). Per migliorare la visibilità dell’ago, la ricostruzione 3D è stata ruotata durante la manipolazione.

**Risultati:**

La modalità di scansione 4D ha permesso il 100 % di visibilità dei tessuti ossei compatti e il 93 % del complesso posteriore. La visualizzazione dell’ago dipendeva fortemente dalla rotazione delle ricostruzioni, con il sensore immobile. L’ago è stato reindirizzato in un paziente (7 %) perché era a contatto con la lamina vertebrale. La dilatazione dello spazio epidurale durante l’iniezione di soluzione salina è stata osservata in cinque pazienti (36 %). In nessun paziente è stato necessario un cambiamento nel livello della puntura; non sono state osservate complicanze associate alla puntura epidurale.

**Conclusioni:**

La navigazione ecografia in 4D potrebbe migliorare l’anestesia epidurale per il miglioramento della risoluzione spaziale offerta all’operatore. La tecnica opera di “contrasto di posizione” deve essere utilizzato per la visualizzazione affidabile dell’ago.

## Introduction

Epidural anaesthesia is widely used in clinical practise [1–6]. However, the success of this method of anaesthesia depends on the ability to correctly estimate the placement of the epidural space to puncture and catheterise it. Currently, the most common method for determining the level of puncture and correct needle direction is based on anatomical landmarks, and the depth of the epidural space placement is assessed by the “loss of resistance” test [7]. The level of puncture as determined by anatomical landmarks is an inaccurate technique, with an error rate as high as 70 % [8]. It can be difficult to determine anatomical landmarks in patients with obesity, indeterminate landmarks, and anatomical variations or anomalies, all of which are factors that can complicate epidural anaesthesia [9]. The technical complexities of the epidural puncture method can increase the risks of neurological complications, unsuccessful blocks, and patient dissatisfaction [10–13].

Ultrasound guidance in nerve blocks is widely used in clinical practice. However, the application of ultrasound during neuraxial anaesthesia has remained limited [14] due to the difficulty of visualising the dense structures surrounding the epidural space and spinal canal [15].

The aims of this study were to identify the optimal technique for navigation and clinical evaluation of 4D (3D real-time mode) ultrasound navigation efficacy during epidural anaesthesia at the lower thoracic and lumbar levels and to assess clinically the efficacy of 4D ultrasound navigation for epidural anaesthesia at these levels.

## Methods

This study was approved by the local ethics committee of First Moscow State Medical University, and written informed consent was obtained from all of the subjects. The study included 16 patients undergoing abdominal surgery.

### Equipment

The ultrasound scan was performed using a Voluson-I (GE HealthCare, USA), equipped with a multi-frequency volume convex transducer of 4–8.5 MHz (RAB4-8-RS). Volumetric scanning was performed by vibration of the linear plane using the transducers (mechanical scanning principle).

### Patients

All the patients received midazolam 0.05–0.07 mg/kg as premedication. Venous access was secured in all of the patients before the epidural puncture for the infusion of crystalloid solutions, and non-invasive monitoring of the patients’ blood pressure, pulse oximetry, respiration rate, and electrocardiogram (ECG) was maintained. The patients were placed in the lateral position.

### Scanning

Before the 4D reconstruction, traditional 2D scanning was performed. The placement of the lumbar epidural was identified by counting up the vertebrae from the sacrum; the thoracic epidural placement was determined by counting down from the twelfth rib. Thereafter, the outlines of the intervertebral joints, vertebral plates, and transverse processes were defined. The dorsal complex was identified by the paramedian oblique access (ligamentum flavum, epidural space, and posterior dura mater), and if possible, the front complex was also determined (anterior dura mater, posterior longitudinal ligament, and vertebral body). Next, a reconstruction frame was constructed. During the 4D reconstruction, tissues with high acoustic impedance (bone structures) and an acoustic window were determined. Because there were no data on the effects of the gel on the neuraxial structures, the transducer was covered with a sterile material to maintain the sterile field; the gel was placed between the transducer surface and the material, and sterile saline, instead of the gel, was applied to the skin.

### Puncture

Two types of needles were used to perform the epidural punctures: Tuohy 18 G needles with ultrasound notches (Tuohy Sono, Pajunk Gmbh) and standard Tuohy 18 G epidural needles (Smith). All attempts at ultrasound scanning and needle insertion were made by one operator. The needle advancement was monitored from two perspectives at the same time: on the sagittal plane in 2D mode, and in volumetric reconstruction mode. The “loss of resistance” test and catheter insertion were conducted by the second anaesthetist. If resistance due to bone contact was encountered during needle insertion, the operator redirected the needle. The number of needle insertions (including needle redirections without removal) necessary to achieve the epidural space in the course of the anaesthesia procedure was counted.

### Anaesthesia

A positive “loss of resistance” test and a negative aspiration test were required before inserting the catheter into the epidural space 5 cm from the tip of the needle in the cranial direction. To avoid intrathecal catheter placement, a standard test dose of lidocaine was infused into the epidural catheter; thereafter, epidural anaesthesia was performed in accordance with the planned operation.

## Data analysis

This study was not powered for statistical analysis. Nevertheless, we calculated the 95 % confidence interval for the probability of success visualisation of needle movement in 4D mode.

## Results

Epidural anaesthesia was performed under 4D ultrasound guidance in 16 patients. The characteristics of these patients are presented in Table 1.

**Table 1:** Characteristics of the patients

Patient number	Age (years)	Sex	Height (cm)	Body mass index	ASA	Puncture level placement	Operation
1	31	M	173	20.0	2	Th11–Th12	Resection of the relegated bowel
2	60	M	174	23.8	3	Th11–Th12	Resection of the sigmoid colon
3	64	M	178	24.6	3	Th12–L1	Resection of the rectum
4	51	M	174	22.8	3	Th11–Th12	Resection of the sigmoid colon and rectum, nephrectomy
5	56	M	175	39.2	3	Th11–Th12	Hernioplasty
6	57	F	150	35.6	2	Th12–L1	Resection of the rectum
7	64	M	178	23.0	2	L1–L2	Sigmostoma surgery
8	65	F	167	21.2	2	Th12–L1	Abdominoperineal resection of the rectum
9	61	F	176	29.1	2	L1–L2	Reconstructive surgery
10	60	M	171	22.6	2	Th12–L1	Reconstructive surgery
11^a^	63	M	167	22.9	3	L2–L3	–
12	23	M	184	29.2	2	L4–L5	Excision of anorectal fistula
13	63	M	165	25.0	2	Th11–Th12	Resection of the sigmoid colon and rectum
14	73	M	170	21.5	2	Th10–Th11	Resection of left parts of the colon
15	59	F	162	31.2	2	Th11–Th12	Laparotomy
16	50	F	160	23.8	2	Th12–L1	Abdomino-anal resection of the rectum, ovary resection

^a^Therapeutic epidural puncture

During 4D scanning, 100 % visualisation of the dense bone structures and 93 % visualisation of the neuraxial structures (the dorsal complex) were accomplished. The “loss of resistance” test was successful in 100 % of cases. The epidural catheter was successfully inserted in 100 % of cases. Some resistance during catheter insertion occurred in one patient. The needle was redirected in one patient (7 %) because it encountered the vertebral bone plate. Dilatation of the epidural space during saline administration was observed in five patients (36 %). The steps of the procedure are presented in Table 2. Needle visualisation strongly depended on the rotation of the reconstructed image while the sensor remained motionless (Table 3).

**Table 2:** Registration of the epidural puncture steps

Patient number	Puncture level placement	Identification of the dense structures	Acoustic window	“Loss of resistance” test	Catheter passing	Number of needle insertions
1	Th11–Th12	Yes	Yes	Yes	Yes	1
2	Th11–Th12	Yes	Yes	Yes	Yes	1
3	Th12–L1	Yes	Yes	Yes	Yes	1
4	Th11–Th12	Yes	Yes	Yes	Yes	1
5	Th11–Th12	Yes	Yes	Yes	Yes	1
6	Th12–L1	Yes	Yes	Yes	Yes	1
7	L1–L2	Yes	Yes	Yes	Yes	1
8	Th12–L1	Yes	Yes	Yes	Yes	1
9	L1–L2	Yes	No	Yes	Yes	1
10	Th12–L1	Yes	Yes	Yes	Yes	1
11	L2–L3	Yes	Yes	Yes	–	1
12	L4–L5	Yes	Yes	Yes	Yes	1
13	Th11–Th12	Yes	Yes	Yes	Yes	1
14	Th11–Th12	Yes	Yes	Yes	Yes	2
15	Th11–Th12	Yes	Yes	Yes	Yes	1
16	Th12–L1	Yes	Yes	Yes	Yes	1
Total		Yes—100 %	Yes—94 %	Yes—100 %	Yes—100 %	1—94 %
		No—0 %	No—6 %	No—0 %	No—0 %	>1—6 %

**Table 3:** Visibility of the needle depending on the viewing angle

Patient number	Needle type	Above view	Side view	Above-side view	“Positional contrast”
1	TuohySono	No	No	Yes	Yes
2	TuohySono	No	Yes	No	Yes
3	TuohySono	No	Yes	Yes	Yes
4	TuohySono	Yes	Yes	No	Yes
5	TuohySono	No	Yes	No	Yes
6	TuohySono	No	Yes	No	Yes
7	Smith	No	Yes	Yes	Yes
8	Smith	No	Yes	No	Yes
9	Smith	No	Yes	No	Yes
10	TuohySono	No	Yes	Yes	Yes
11	TuohySono	Yes	No	Yes	Yes
12	Smith	No	Yes	No	Yes
13	Smith	No	Yes	No	Yes
14	Smith	No	Yes	No	Yes
15	Smith	No	No	No	Yes
16	Smith	No	No	No	Yes
Total		Yes—13 %	Yes—75 %	Yes—31 %	Yes—100 %
		No—88 %	No—25 %	No—69 %	No—0 %
95 % CI for proportion of success		0—29 %	54—96 %	9—54 %	85—100 %

None of the patients required changes in the level of puncture placement, and no perforations of the dura mater or any other complications related to the epidural puncture were noted. The clinical efficacy during the procedure was sufficient; none of the patients had any complications associated with the epidural anaesthesia technique.

## Discussion

There are limited data about real-time ultrasound guidance, i.e., when the needle is inserted “in-plane” with the ultrasound beam [16–18]. This method has two limitations: first, the two-dimensional (2D) image obtained provides data about a very restricted puncture area; and second, the needle must be directed along the narrow plane of the ultrasound beam (1–2 mm). Thus, to realise the potential of this method fully, enhanced requirements for performing blockade techniques and personal experience must be employed. The dimensional representation of the image can improve orientation in the anatomical zone of interest, including guiding of the needle position in space.

Only one pilot experimental study assessing the potential of 4D ultrasound (3D real-time mode) has previously been conducted. The authors demonstrated the concept of vertebral structure sonography with dimensional reconstruction, but the needle visualisation, and thus real-time guidance, was complicated. Among their reasons for these complications, the authors listed the embalming of the corpses used, the high density of the surrounding structures, and the low transducer resolution [19].

**Fig. 1:**
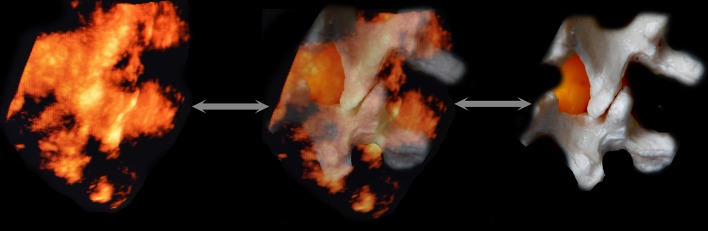
Paramedian sagittal sonogram of the lumbar spine. Dense bone structures look on volumetric ultrasound like bright surfaces, if the reflection of the ultrasound beam returns to the sensor directly. Conversely, the spinous processes, located at an obtuse angle or parallel to the US beam, look like anechoic recesses. Adjusting the signal processing program can find an acceptable balance between reflection from dense structures and incidental noise filtering (fat, ligament, muscle, etc.)

Optical mapping structures in 4D reconstruction are based on the layers of the scanned 2D images and, therefore, they do not fundamentally differ in interpretation. The border of tissue (e.g., bone/muscle) perpendicular to the sensor appears as a hyperechoic area (lamina, articular and transverse processes). Areas located at an acute angle are visualised slightly less clearly. Areas lying at an obtuse angle relative to the sensor could appear as anechoic areas (spinous processes) (Fig. 1). In contrast to traditional 2D ultrasound, with which only a hyperechoic strip is visible, and the operator must form a coherent picture virtually moving the sensor on the skin on the transverse plane, with 3D imaging, volumetric areas appear. Because 3D (4D) modelling consists of mathematical processing of a multitude of dimensional images, for the best results, good 2D image scanning should be achieved, and the appropriate soft program should be used. The front and dorsal complexes appear as hyperechoic structures located under the lamina. The size of these structures could predict the size of the acoustic window and, therefore, the complexity of the puncture of the selected interval. The resolution of the equipment did not allow for differentiation of the equipment structures included in the dorsal (the ligamentum flavum and posterior epidural space of the dura mater) or especially the frontal system (front dura of the posterior longitudinal ligament and the posterior surface of the vertebral body).

**Fig. 2:**
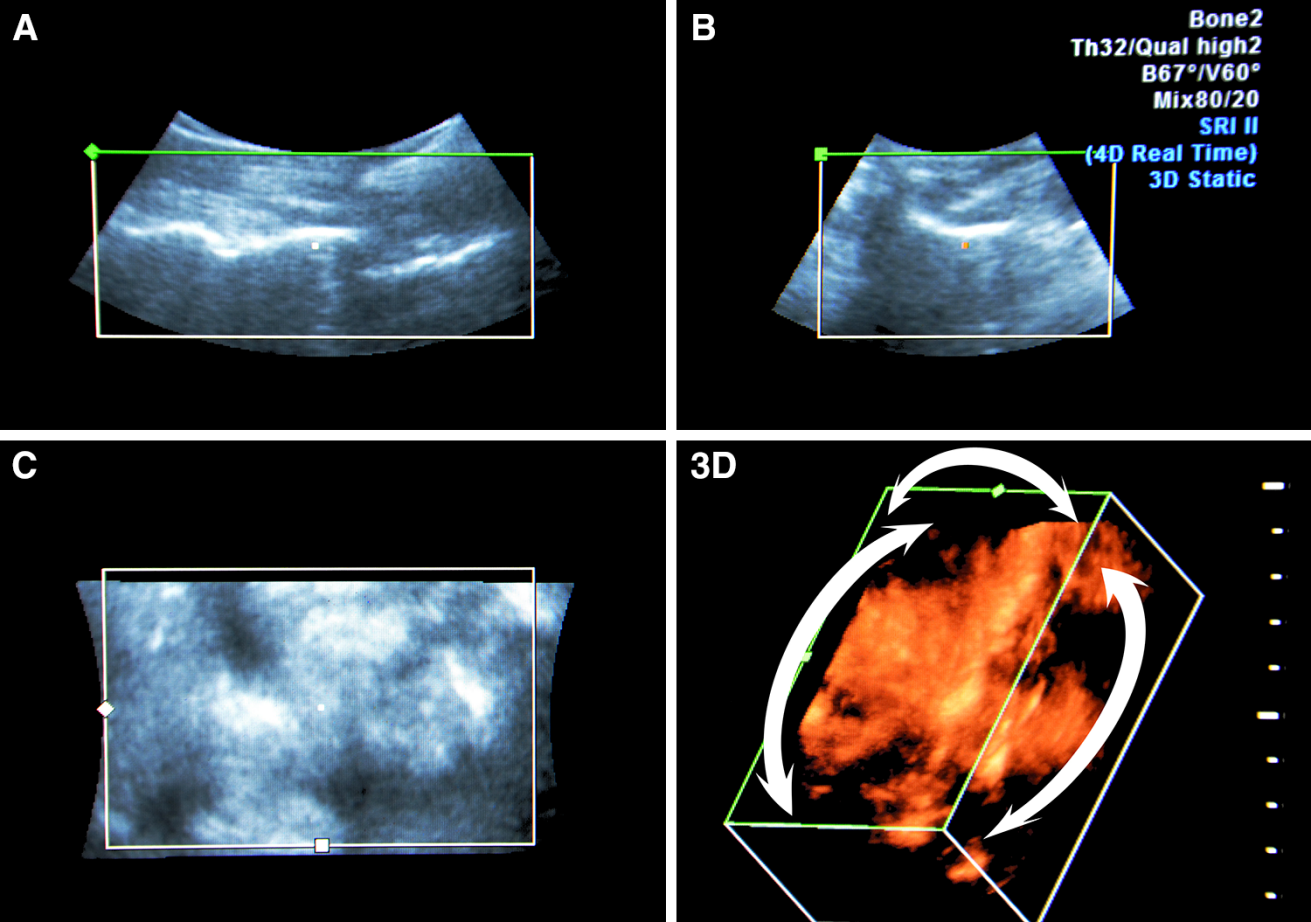
Paramedian sagittal sonogram of the lumbar spine. *A* is the Longitudinal paramedian image, *B* is the axial image, *C* is the coronal image, and *3D* is the three-dimensional reconstruction with frame reconstruction (set by the operator). Note that the *3D* image can rotate at any angle (*white arrows*), and the location and image slices *A*, *B*, and *C* remain unchanged. Additionally, the operator can change the viewing scale of the image to see the details and improvements or, in contrast, zoom out to encompass the whole panorama

**Fig. 3:**
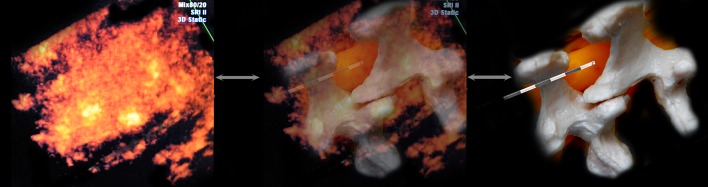
View of epidural puncture from above. The needle is inserted in a right paramedian approach. The *top view* allows for control of the position of the needle relative to the spinous and articular, transverse processes. Until further advancement of the needle affords the opportunity to make corrections, the needle is directed laterally or medially

**Fig. 4:**
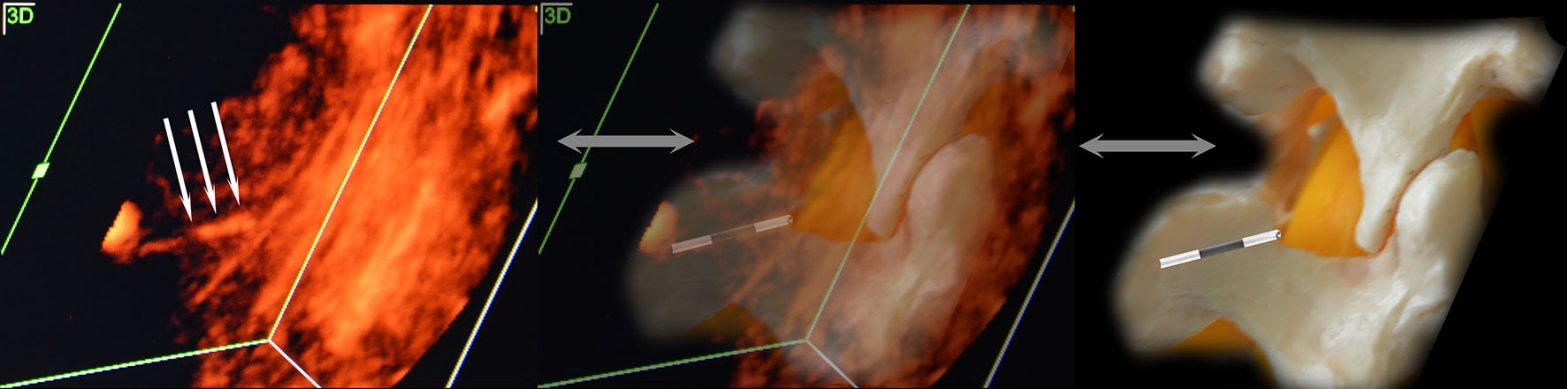
View of epidural puncture from the above side. The needle’s hyperechoic shadow is superimposed with the acoustic shadow of the spinous process. On this image, the expansion of the acoustic shadow of the needle can be seen (*white arrows*). This effect is a special needle, marked “cornerstone” at the tip, and although it creates an optical illusion of thickening of the needle, it could help to convince the operator, once again, of the position of the needle tip. This process can be useful when there is insufficient image quality or during the process of mastering the technique

**Fig. 5:**
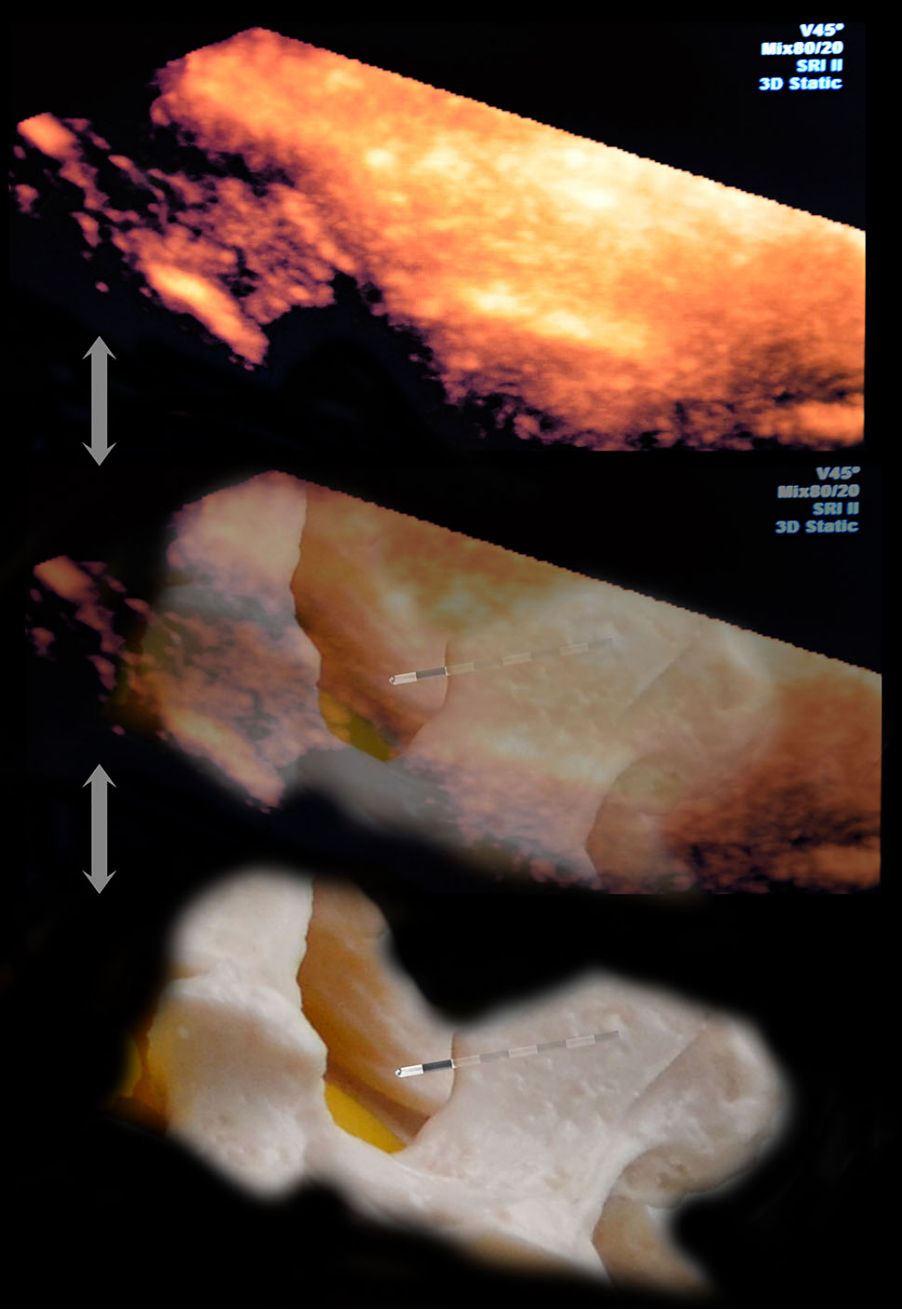
Medial view of epidural puncture. Rotation and scaling of the image to the interval of interest can be considered the complex rear (seen as a hyperechoic stripe), the hypoechoic spinal space filled with cerebrospinal fluid and, in some cases, the front complex (the hyperechoic strip located deeper). At this stage, we were not able to separate the individual elements of the rear of the complex. With slow careful moving of the needle forward from this position, we could see the moment of contact of the needle tip with the rear complex (the epidural space). In some cases, it was also possible to control the expansion space when saline was injected

An essential benefit of 3D modelling, except for volumetric representation, is that it can rotate an image at any angle, approximate and move away it, and focus on areas that are of interest at each time point, without changing the position of the transducer and needle (Fig. 2). This benefit allows the sensor not to follow the needle, to move over the skin, and to choose the most beneficial foreshortening at every moment for better control of the needle tip. We noted from this perspective that regarding the image of the needle, seen as a hyperechoic strip superimposed on the underlying hyperechoic formation, its visibility could deteriorate significantly. Therefore, for reliable control of the needle in the space, the operator should dynamically change the angle and zoom navigation. To clarify the position of the needle relative to the spinous and articular processes, the “above view” is optimal (from the sensor, Fig. 3). Then, to verify the depth of the needle passing through the tissues, until it is near the interlaminar space, the above-side view is optimal, with rotation of the image so that the needle’s hyperechoic shadow is superimposed on the acoustic shadow of the spinous process, which clearly stands out as a relatively large anechoic structure (Fig. 4). When approaching a planned interval, control of the needle tip from this angle is difficult. For maximum detail, the operator should reasonably rotate the image on the control from the medial side, “through the spinous process”, focusing the view on the target interspace (Fig. 5). From this position, the operator can see and directly contact the needle tip with the dorsal epidural space. Thus, the method of “positional contrast”—the dynamic change in viewing angle of reconstruction in real time for the best visual inspection of the needle position, combining the hyperechoic shadow of needle with underlying relatively hypoechoic forms—is optimal for puncture control during each stage.

In our study, an above view was successful in 30 % of cases, and the technique of “dynamic contrast” was effective in 85–100 % of attempts (Table 3).

To perform anaesthesia, the “Epi Long Soft Sono Set” (Pajunk Gmbh) and “Minipack” (Smith) sets for epidural anaesthesia were used. The “Minipack” consists of an 18 G Tuohy needle and a polyether block amide 110-cm catheter. The “Epi Long Soft Sono Set” is a TuohySono needle set intended for ultrasound guidance. The distal end of the needle has special reflectors that are designed to reflect ultrasonic waves with maximum intensity under different needle tilts to improve ultrasound visualisation of the needle. A catheter reinforced with a steel wire to protect against inflection was also used with these needles.

The notched needles with improved ultrasound beam visibility had the same visibility over their entire lengths as the Tuohy needles. Although the circularly arranged incisions on the needle created the optical illusion of needle thickening in some cases, they provided better control of the depth of needle insertion (Fig. 4).

The epidural space is filled with adipose tissue, and it has low acoustic impedance, which was decreased after saline administration. Despite using a reinforced catheter, attempts to visualise the catheter passing into the accessible part of the epidural space were unsuccessful. This inability was potentially due to the insufficient resolution power of the device, the small number of observations, or operator inexperience with dimensional ultrasound.

The need for the participation of two anaesthesiologists at the initial stage of development of the methods was dictated by the necessity of reliable verification of the epidural space with the traditional method (LOS). In the absence of a special device ensuring the identification of the epidural space [17], one anaesthesiologist cannot perform gentle advancement of the needle, control on 4D imaging for device reliability, and LOS. Participation of a specialist will be possible to consider further enhanced navigation by ultrasonic techniques and the use of special devices to facilitate puncture.

## Conclusion

4D ultrasound guidance can facilitate epidural anaesthesia by improving the dimensional orientation of the operator. For reliable needle visualisation, a “positional contrast” method should be used, and special needles for ultrasound guidance can facilitate the control of the depth of needle insertion. Further studies are required to assess the efficacy of dimensional ultrasound guidance during neuraxial blockade in patients with expected difficulties, such as obesity or altered anatomy.

## Electronic supplementary material

Below is the link to the electronic supplementary material.

Supplementary material 1 (MP4 5394 kb)

Supplementary material 2 (MP4 7126 kb)

Supplementary material 3 (MP4 9420 kb)
